# Simple Congenital Hamartoma of the Retinal Pigment Epithelium

**DOI:** 10.18502/jovr.v15i2.6747

**Published:** 2020-04-06

**Authors:** Austin S. Nakatsuka, Touka Banaee, Emma Loucks, Jaafar El-Annan

**Affiliations:** ^1^ Department of Ophthalmology and Visual Sciences, University of Texas Medical Branch, Galveston, Texas, USA; ^2^ Department of Ophthalmology, Mashhad University of Medical Sciences, Mashhad, Iran; ^3^ University of Texas at MD Anderson, Houston, Texas, USA; ^4^ Blanton Eye Institute, Houston Methodist Hospital, Houston, Texas, USA

##  PRESENTATION

**Figure 1:**
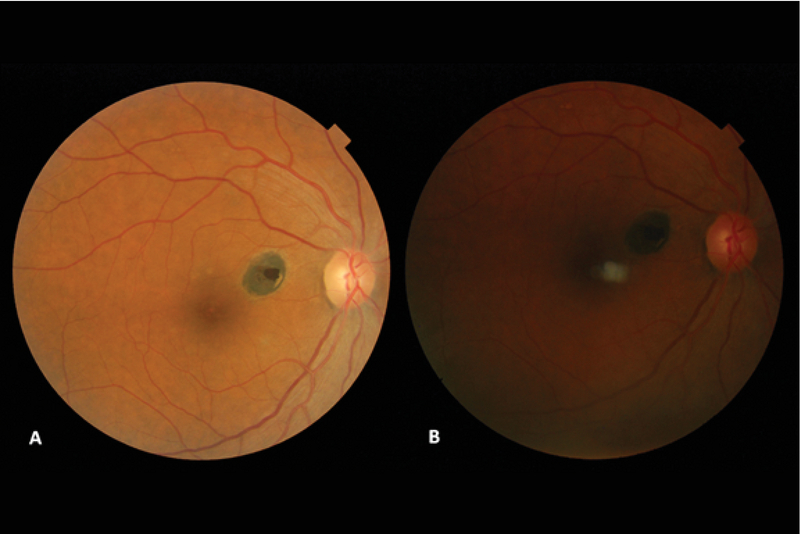
Fundus photographs showing a simple congenital RPE hamartoma with no change from 2007 (A) to 2017 (B).

An asymptomatic 63-year-old woman presented for annual ophthalmic examination at University of Texas Medical Branch eye clinic and was found to have a simple congenital hamartoma of the retinal pigment epithelium (RPE) in the left eye. The best corrected visual acuity was 20/20 and the visual field was within normal limits. On fundus examination, a circular hyperpigmented retinal lesion measuring less than the diameter of an optic disc was detected superonasal to the fovea. Old records indicated that the lesion had been present since at least 12 years before, showing no significant morphological changes on serial fundus photographs [Figures 1A and 1B].

**Figure 2:**
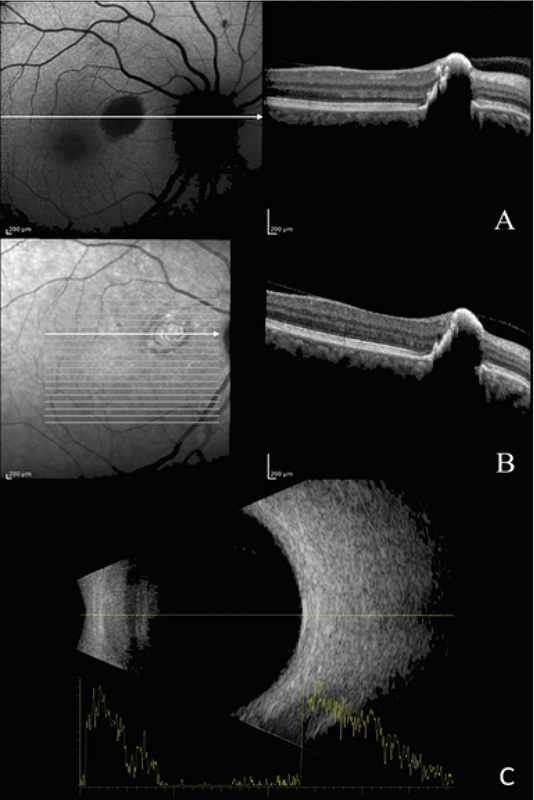
Optical coherence tomography (OCT) showing a retinal lesion with high surface reflectivity, deep optical shadowing, and full-thickness retinal involvement. Image (A) was taken in 2009, and image (B) in 2017. The arrows on the left side autofluorescence (AF) and infrared images show the level of B scans that although not registered pass through the same location based on fundus landmarks. The lesion shows hypoautofluorescence in AF imaging. (C) B scan ultrasonography reveals a flat lesion with high internal reflectivity.

The lesion showed hypoautofluorescence in autofluorescence imaging [Figure 2A] and optical coherence tomography (OCT) revealed high surface reflectivity with deep optical shadowing and full-thickness retinal involvement that had been stable over time [Figures 2A and 2B]. B-scan ultrasonography revealed a flat lesion showing medium to high internal reflectivity [Figure 2C].

##  DISCUSSION

Simple congenital hamartoma of the RPE is a benign tumor composed of a hyperplastic RPE with variable vascularity and is often discovered incidentally in asymptomatic children and young adults.^[1,2]^ It is a rare tumor and presents as a pigmented mass protruding from the RPE into the retina and at times into the vitreous cavity, usually at the posterior pole.^[1,2]^ Shields et al^[2]^ reported the presence of a minimally dilated feeding retinal arteriole and a draining venule in five cases. We did not observe this finding in the present case.

According to previous studies, the retinal tissue surrounding the hamartoma may show minimal signs of traction,^[3]^ which was not present in the current case. These lesions showed early hypofluorescence in fluorescein angiography with some variable hyperfluorescence seen in later frames due to either RPE atrophy or intrinsic tumor vasculature.^[2]^ OCT features of the lesion show a hyper-reflective surface and dense shadowing.^[4]^ Simple hamartomas of RPE are usually small and cannot be easily seen by ultrasonography. If visible, they have medium to high internal reflectivity on ultrasound.^[2]^

Differential diagnoses include combined hamartoma of the retina and RPE, adenoma or adenocarcinoma of the RPE, congenital hyperplasia of the RPE, choroidal nevus, choroidal melanoma, RPE hyperplasia, and intraocular foreign body.^[2]^ The absence of a history of trauma, retinal traction, time-related growth features, and the typical funduscopic features of the lesion can help differentiate between these diagnoses.

The present case is particularly interesting because the stability of lesion is documented by fundus photographs spanning a duration of 10 years and by OCT images that are 8 years apart.

##  Financial Support and Sponsorship

Nil.

##  Conflicts of Interest

There are no conflicts of interest.
